# Thin-Walled Double Side Freeform Component Milling Process with Paraffin Filling Method †

**DOI:** 10.3390/mi8110332

**Published:** 2017-11-17

**Authors:** Jun Zha, Jing Chu, Yipeng Li, Yaolong Chen

**Affiliations:** State Key Laboratory for Manufacturing Systems Engineering, Xi’an Jiaotong University, Xi’an 710000, China; jun_zha@xjtu.edu.cn (J.Z.); chujing3321@gmail.com (J.C.); liyipeng@mail.xjtu.edu.cn (Y.L.)

**Keywords:** thin-walled double side, freeform surface, filling method

## Abstract

The machining of thin-walled double side freeform component has many challenges in terms of the geometrical complexity, high-requirement accuracy, and especially low stiffness. This paper surveys the filling method during the milling processes of thin-walled double side freeform component. Firstly, the DEFORM-3D was used to analyze and calculate the surface residual stress which provides a theoretical basis for parameters selection of the rough milling process, and the optimal milling parameters were obtained by the Taguchi method. Residual stress measurements have been carried out to verify the simulation results. The results show the difference between simulation and experimental data is less than 15%. Secondly, semi-finishing parameters and finishing process parameters were determined by equal error step length and step distance method. Thirdly, two machining experiments were conducted with and without paraffin filling, and the accuracy was measured by coordinate measurement machine. The results shown that the PV values are 25.16 μm and 20.34 μm for the concave and convex surface, and the corresponding RMS values are 13.75 μm and 11.93 μm in the first milling experiment. The PV values have improved to 8.53 μm and 7.12 μm, and RMS values have improved to 2.45 μm and 3.05 μm by the filled method applied.

## 1. Introduction

Due to the geometrical complexity and high-requirement accuracy of freeform surface, the machining and measurement are still a common challenge in industry [1,2,3]. Successful machining of aspheric surface not only relies on the high precision of machine tools, but also largely depends on the measurement method [3]. Machining for single side aspheric surface has been investigated by many researchers [1,2,3,4], and lots of valuable approaches have been proposed. For the characterization of form accuracy and surface quality of single side freeform surfaces, there is still lack of specific standards and techniques [5]. In general, for the inspection of the single side freeform surface, the most important instruments are coordinate measuring machines with high accuracy equipped with contacted scanning or non-contacted method [4,6].

According to the abovementioned research status, it can be found out that studies on double side aspheric components are rarely reported in literature, especially in the machining and accuracy measurement areas. The thin-walled double side freeform surface component is a comparatively good means of achieving effective control of the laser beam, and has various applications such as laser beam shaping [7,8], light-emitting diode uniform illumination [9,10], narrow illumination [11], and heads up display in the aircraft. However, the clamping method during the machining and the accuracy measuring method always affect surface quality of the thin-walled double side freeform components. The milling force, heat, and clamping force during the machining processes will have negative effects on form accuracy and surface quality of the thin-walled component due to the low stiffness and strength characteristics. In addition, the induced vibration will produce chatter marks on the component. Therefore, a filling method by auxiliary supporting technology for improving the stiffness and hence the machining accuracy of the thin-walled double side freeform component during the milling processes was studied in this research.

## 2. Thin-Walled Double Side Aspheric Component Model

To describe an aspheric lens with a symmetrical optical axis of *z*, the curved surface of the lens with respect to the position axis *x*, *y*, which is perpendicular to the optical path can be expressed as

(1) $$z=\frac{c_{x}x^{2}+c_{y}y^{2}}{1+\sqrt{1-(1+k_{x})c_{x}^{2}x^{2}-(1+k_{y})c_{y}^{2}y^{2}}}$$

In Equation (1), the radius curvature at the vertex of the aspheric curve are defined as *r_x_*, *r_y_* and the eccentricity are as *e_x_* and *e_y_*. The expression of *c_x_*, *c_y_*, *k_x_*, and *k_y_* are given as

*c_x_* = 1/*r_x_*, (2)

*c_y_* = 1/*r_y_*, (3)

(4) $$k_{x}=1-e_{x}^{2},$$

(5) $$k_{y}=1-e_{y}^{2},$$

**Figure 1:**
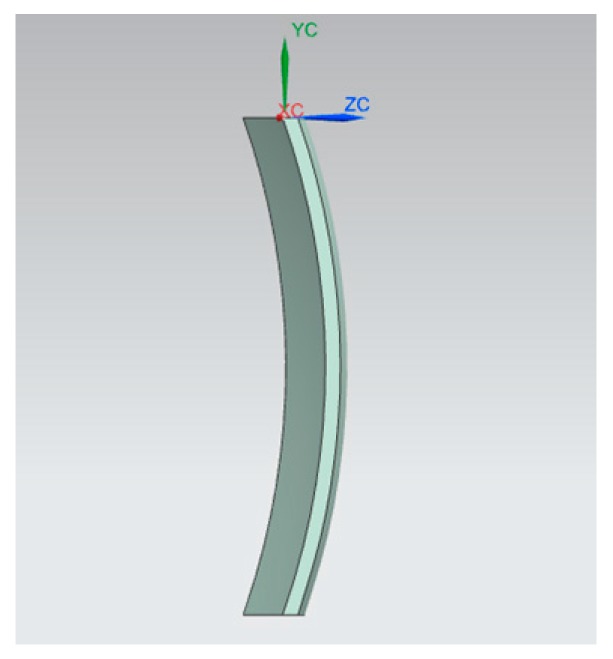
Thin-walled double side aspheric component model.

According to the described function, the model was given as Figure 1 shown.

## 3. Simulation and Experiment with the Taguchi Method

The Taguchi method is an efficient and systematic approach that can reduce the experimental trials necessary to determine the optimal conditions [12]. Previous studies have also been conducted in the optimization of cutting parameters for turning operations [13], end milling operations [14], and the grinding processes [15]. In this research, the 6061-T651 aluminum alloys instead of K9 glass was used to manufacture the thin-walled double side aspheric surfaces. In this section, the rough machining parameters optimization was conducted by Taguchi method, and the effects of four control factors on the residual stress along *xy*-plane of the thin-walled double side freeform component were analyzed and quantified.

### 3.1. Milling Parameters Optimization by Taguchi Method

The ratio of the wall thickness to radius is less than 1:30. The milling force, clamping force, milling heat, and the effect of initial residual stress will cause the thin-walled workpiece to deform easily. So the milling force, temperature distribution, and the residual stress in the process of rough machining are of great importance to the form accuracy.

The DEFORM-3D was used to analyze and calculate residual stress in the rough milling process, provides a theoretical basis for parameters selection of the rough milling process. In the simulation, four parameters—namely spindle rotation speed (*n*), feed engagement (*f_z_*), milling depth (*a_p_*), and milling width (*a_c_*)—are determined as the control factors which mainly affect the residual stress in the rough milling process. Four control factors and their levels are listed in Table 1.

### 3.2. Residual Stress Measurement

The experiment based on *L*_16_ (4^5^) was conducted. Residual stress along the *xy*-plane on the surface of the workpiece was measured with *X*-ray method.

### 3.3. Analysis and Machining Parameters Determination

**Figure 2:**
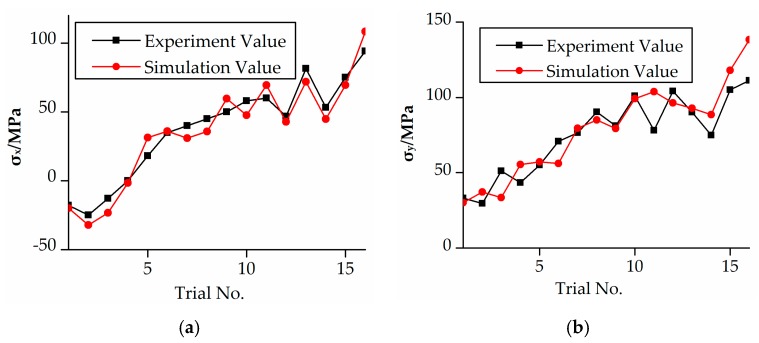
Comparison of simulation and experiment results. (**a**) *X*-direction; (**b**) *Y*-direction.

The residual stress along the *xy*-plane on the surface of the workpiece were obtained by simulation and experiment, as shown in Figure 2. It can be seen that the simulation results coincided basically with the experiment results. The difference is within 15%, and it was the comparison between the simulation and average experimental residual stresses. It was due to the cutting tool having a large force induced deformation when large machining parameter values were adopted, like the milling depth and feed engagement increased, which affected the residual stress values.

**Figure 3:**
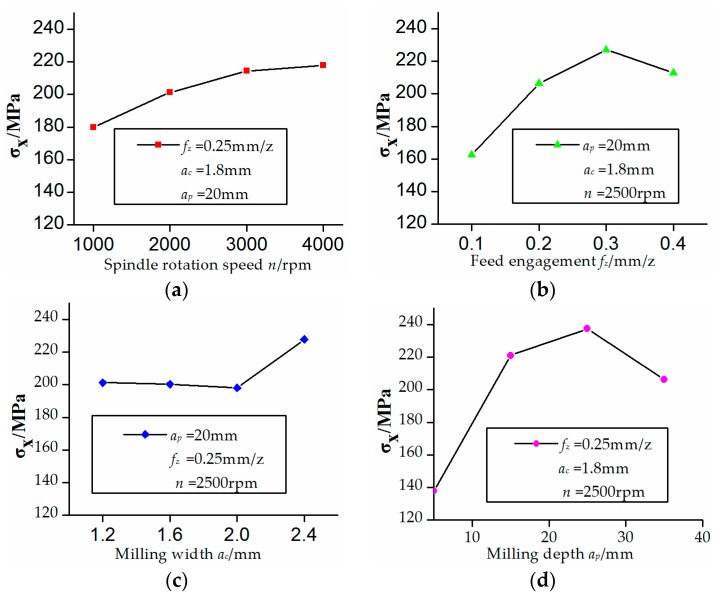
Effects of control factors on *X*-direction residual stress. (**a**) Relationship between spindle rotation speed and residual stress; (**b**) Relationship between feed engagement and residual stress; (**c**) Relationship between milling width and residual stress; (**d**) Relationship between milling depth and residual stress.

**Figure 4:**
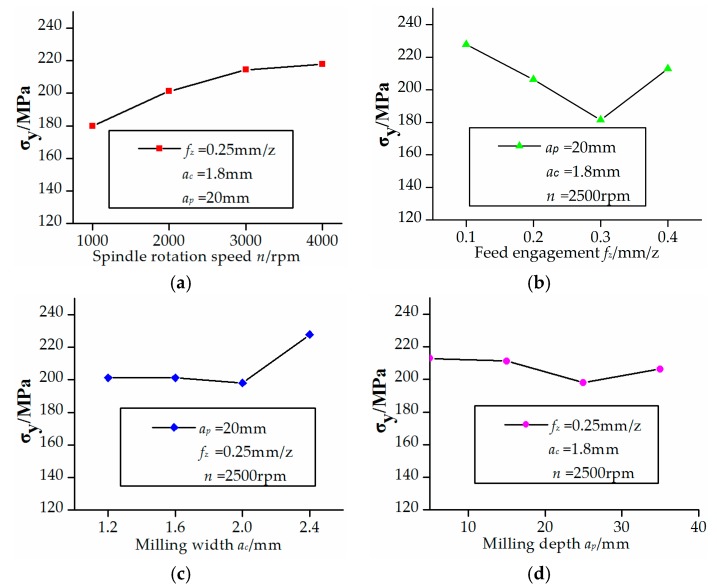
Effects of control factors on *Y*-direction residual stress. (**a**) Relationship between spindle rotation speed and residual stress; (**b**) Relationship between feed engagement and residual stress; (**c**) Relationship between milling width and residual stress; (**d**) Relationship between milling depth and residual stress.

The effects of four control factors on the residual stress along the *xy*-plane were shown in Figure 3 and Figure 4. It can be found by analysis of variance that the milling depth and feed engagement have the most effects on residual stress on the *xy*-plane followed by spindle rotation speed and milling width. The residual stress was produced by milling heat stress. The component surface residual stress increased as the milling depth increased due to the increasing of the milling force. Also, the residual stress increased as the feed engagement increased due to the friction heat between the cutting tool and the workpiece.

Taking the effects of four milling parameters on residual stress and the machining stability into consideration, the rough milling parameters (*n* = 3000 rpm, *f_z_* = 0.2 mm/z, *a_c_* = 1.1 mm, machining allowance is 1 mm) were determined ultimately.

## 4. Machining Processes for Freeform Surface

### 4.1. Experiment Setup 1

**Figure 5:**
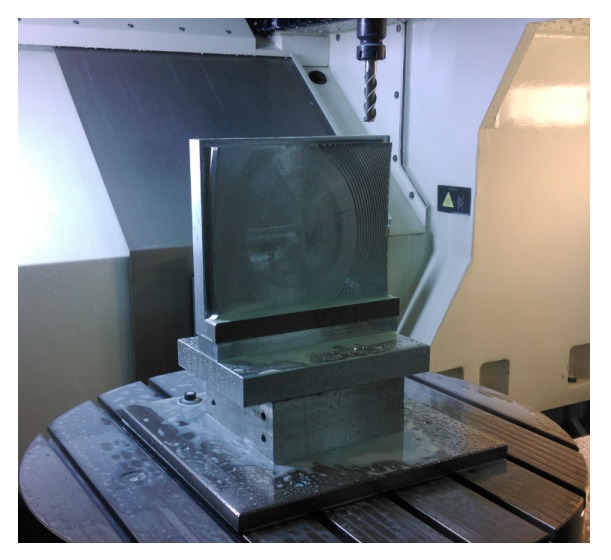
Experimental setup.

The workpiece was machined by a five-axis precision machining center DMG HSC75 Linear (DMG, Bielefeld, Germany), the stroke range along *x*, *y*, and *z* axes are 750, 600, and 560 mm, respectively. The rotational range of *C*-axis is 360° and the rotational range of *B*-axis is −10~110°. Repositioning accuracy for three linear axes and two rotation axes were 3 μm and 5 arcseconds, respectively. Figure 5 shows the configuration of the experimental setup, where the workpiece was fixed on the rotation table. The machining of double freeform surface was completed in a single set-up for avoiding the positioning error due to the repeated clamping processes.

In the experiment, rough machining process parameters were determined in the above section. Equal error step length and step distance were adopted to determined semi-finish machining process parameters (*n* = 12000 rpm, *f_z_* = 0.3 mm/z, *a_c_* = 0.2 mm, *a_p_* = 0.2 mm) and finish machining process parameters (*n* = 12000 rpm, *f_z_* = 0.125 mm/z, *a_c_* = 0.05 mm, *a_p_* = 0.05 mm). Semi-finish machining process was implemented by hierarchical processing. The machining allowance for the first layer is 0.3 mm and the second layer is 0.05 mm.

### 4.2. Experiment Result 1

**Figure 6:**
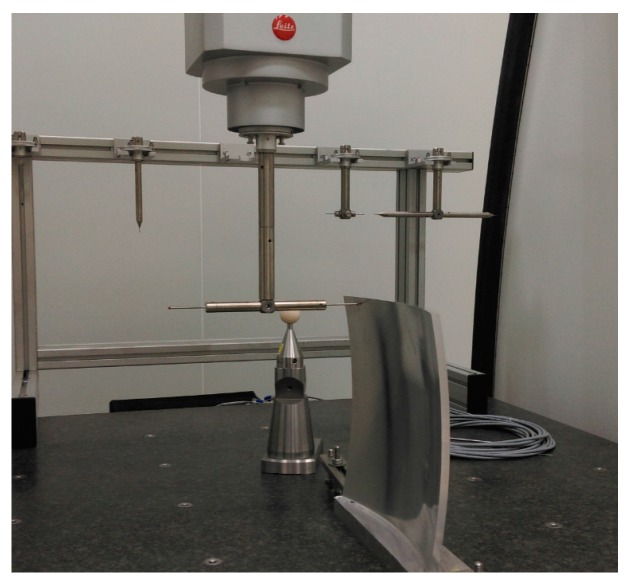
Measurement of form accuracy.

**Figure 7:**
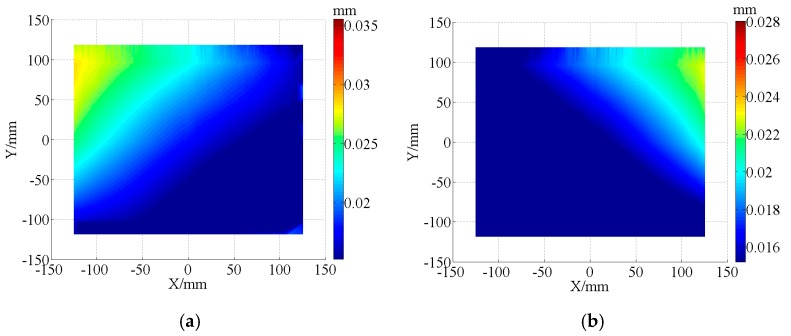
Error distribution by contacted scanning method. (**a**) Error distribution of concave surface 1; (**b**) Error distribution of convex surface 1.

The form accuracy of the freeform component was measured by an ultra-precise three-coordinate measuring machine for which measurement error in any position of measurement range is within (0.3 + *L*/1000) μm using contacted scanning method. Then, the measurement of double freeform surface was completed in a single set-up, as shown in Figure 6. The PV values are 25.16 μm and 20.34 μm for concave and convex surfaces, respectively. The corresponding RMS are 13.75 μm and 11.93 μm. The measurement results were shown in Figure 7.

## 5. Experiment with Paraffin Filled Method

In the comparison experiment, paraffin filling method was adopted, which the material melted and shrank easily, and will not produce stress during perfusion and solidification processes for the thin-walled parts, and not shed off or dissolve in the cutting fluid during machining. The paraffin can be removed easily by acetone and no deformation occurred. This can increase the rigidity of the workpiece and avoid vibration caused by lack of support.

### 5.1. Experiment Setup 2

**Figure 8:**
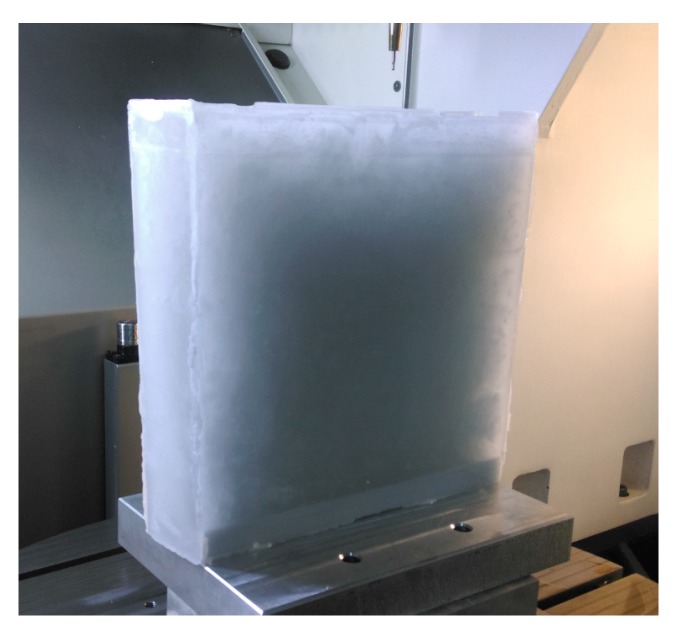
Workpiece with paraffin filling.

**Figure 9:**
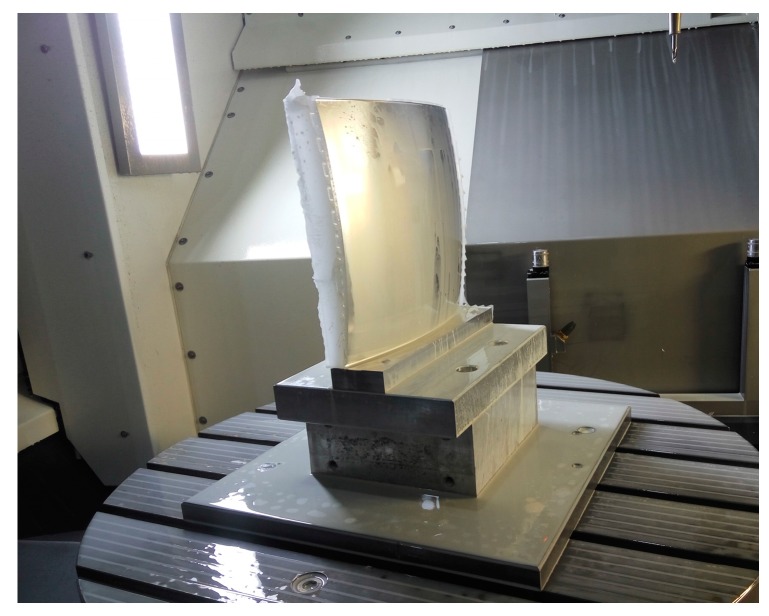
Freeform component after one surface machining was completed.

In the experiment, the workpiece was filled with paraffin after a rough machining process, as shown in Figure 8. The paraffin on one freeform surface was machined with a thickness 2 mm. After the finish process of one freeform surface, the workpiece is refilled with paraffin. Then, the paraffin on the other freeform surface was also machined with a thickness 2 mm. Semi-finish and finish machining process of the other freeform surface were completed, as Figure 9 shows. The whole process will completed after the paraffin removed. The parameters adopted in the experiment with paraffin filling were same as the experiment without paraffin filling.

### 5.2. Experiment Result 2

**Figure 10:**
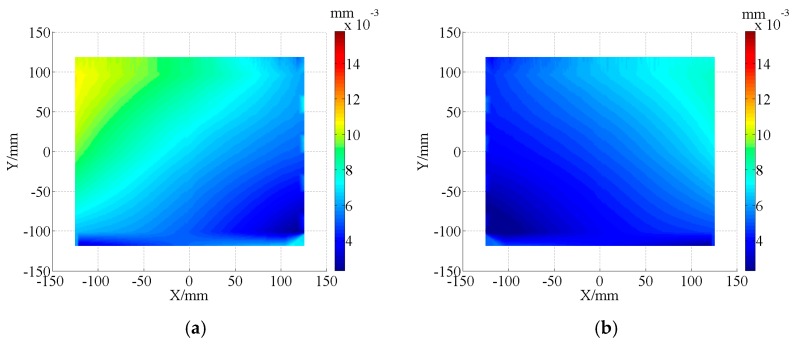
Error distribution of the freeform component machining with paraffin filled. (**a**) Error distribution of concave surface 2; (**b**) Error distribution of convex surface 2.

Form accuracy of the thin-walled double side freeform component was measured by the same method mentioned at Section 4.2. The PV values are 8.53 μm and 7.12 μm for concave and convex surface, respectively, and the error distribution was shown in Figure 10. The corresponding RMS values are 2.45 μm and 3.05 μm.

## 6. Discussions

It can be found that the stress is basically residual tension stress after the rough milling processes, as shown in Figure 2. It increased as the spindle rotation speed, milling depth, milling width, and feed engagement increased. Which indicated the established residual stress simulation model can be properly used to analysis the effects of milling parameters on residual stress. The simulation results of Trials 1–13 were in good agreement with the milling experiment results. However, simulation results and milling experiment results have been much more varied for Trials 14–16, especially on the *y*-axis. This is mainly caused by the large values for the machining parameters and the milling direction in the experiment.

The thin-walled double side freeform component has low stiffness during the semi-finishing and finishing machining processes, make processing precision not easily guaranteed. The auxiliary support material with liquid–solid transition and easily removed characteristics can be suitable for achieving flexible fixture fabrication for increasing the stiffness of thin-walled components. Depending on the comparison between the measurement results, as shown in Figure 6 and Figure 9, the PV values significantly improved by 66.1% and 65.0%, the RMS values improved by 82.2% and 74.4% for concave and convex surfaces of the thin-walled double side freeform component, by the paraffin filled method applied.

## 7. Conclusions

In this paper, detailed process for machining the freeform surface using a five-axis precision machining center were described, and the optimization of machining parameters for the milling process using the Taguchi method were performed. The main results are as follows:

(1) Four operational parameters of spindle rotation speed (*n*), feed engagement (*f_z_*), milling depth (*a_p_*), milling width (*a_c_*) were selected as control factors to perform the Taguchi approach. Experimental trials based on the L_16_ (4^5^) were carried out. The milling depth and feed engagement have the most effects on residual stress.
(2) The PV values 25.16 μm and 20.34 μm could be significantly improved to 8.53 μm and 7.12 μm, and the RMS values 13.75 μm and 11.93 μm could be significantly improved to 2.45 μm and 3.05 μm, by the paraffin filling method applied for the machining of thin-walled double side freeform component.

## Figures and Tables

**Table 1:** Control factors and their levels of rough milling process.

No.	*n* (rpm)	*f_z_* (mm/z)	*a_p_* (mm)	*a_c_* (mm)
1	1000	0.1	5	1.2
2	2000	0.2	15	1.6
3	3000	0.3	25	2
4	4000	0.4	35	2.4
